# Psychiatric symptoms in general population and health personnel during the second and third waves of COVID-19 in Mexico

**DOI:** 10.1192/j.eurpsy.2023.886

**Published:** 2023-07-19

**Authors:** M. S. González, S. Luna, A. B. Cuéllar

**Affiliations:** Psychiatry, Universidad Autónoma de Nuevo León, Monterrey, Mexico

## Abstract

**Introduction:**

The COVID-19 pandemic has significantly affected mental health. However, its impact between different pandemic waves and different populations has been scarcely studied.

**Objectives:**

The aim of this study was to analyze the differences in psychiatric symptomatology between the general population (GP) and health personnel (HP) during the second and third waves of COVID-19 in Mexico.

**Methods:**

404 participants were included as part of a cross-sectional study conducted during the COVID-19 pandemic, using an online survey. Second wave covered from September 27, 2020 to April 17, 2021 and the third wave covered from June 6, 2021 to October 23, 2021. GP refers to Mexican residents during the pandemic, and HP includes healthcare workers (doctors, nurses, residents). Sociodemographic data were collected and scales of depression (Patient Health Questionnaire 9), anxiety (General Anxiety Disorder -7), insomnia (insomnia severity index), and post-traumatic stress (Impact of event scale revised) were applied. We gather information in a database in Excel, for later analysis using IBM SPSS Statics 21. Traditional descriptive statistics for quantitative variables and frequencies for qualitative variables were obtained. Association and statistical correlation were analyzed using Chi2 tests.

**Results:**

71.3% of the collected sample were female, mean age 35.5 (sd= 11.6), the 62.5% consisted of health personnel, the majority were single 48.9%, with postgraduate education 48.9%, middle class (97.2%). A higher percentage of symptoms of depression and anxiety was observed in health personnel compared to the general population during the second wave of COVID-19 (33.9% vs. 19.5%, p=0.047; 18.2% vs. 39.3%, p=0.006). However, during the third wave of COVID-19, more depressive, anxious and insomnia symptoms were observed in the general population compared to health personnel (73.9% vs. 44.4%, p=0.020; 73.9 vs. 25.9%, p= 0.000; 43.5% vs. 11.1%, p=0.008) (Figure 1).

**Image:**

**Figure:**
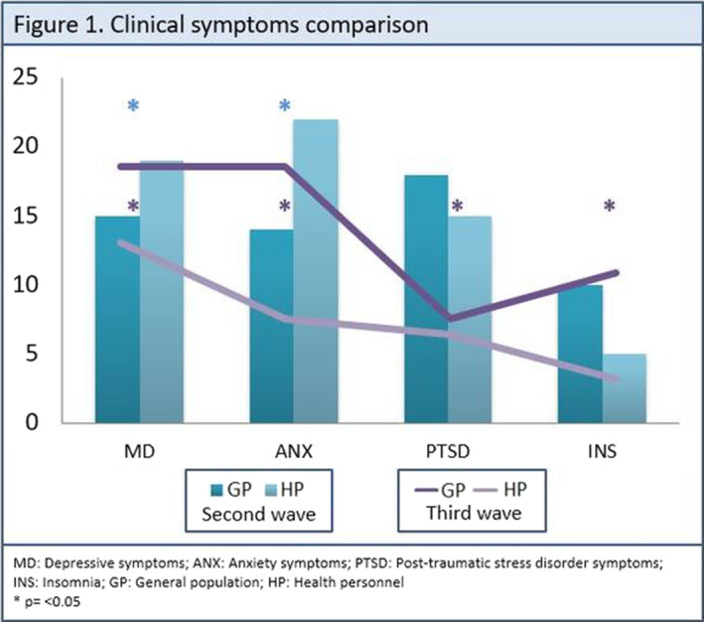

**Conclusions:**

Health personnel presented more depressive and anxious symptoms during the second wave of COVID-19 compared to the general population, however, the results were inverse during the third wave, showing more psychiatric symptoms in the general population with significant differences. This may be due to various factors, including unawareness, fear of the disease, and exposure during the second wave of the pandemic of health personnel. Moreover, long-lasting containment measures could have overwhelmed the GP by the third wave. Our study underscores the importance of addressing HP mental stressors to increase its resilience in similar health crises.

**Disclosure of Interest:**

None Declared

